# Rehabilitation Protocols and Functional Outcomes in Oncological Patients Treated with Modular Megaprosthesis: A Systematic Review

**DOI:** 10.3390/cancers17182951

**Published:** 2025-09-09

**Authors:** Filip Fryderyk Brzeszczyński, Michał Karpiński, Marcel Aleksander Brzeszczyński, Oktawiusz Bończak, David F. Hamilton

**Affiliations:** 1 Department of Trauma, Orthopaedics and Musculoskeletal Oncology, Copernicus Memorial Hospital, 93-513 Lodz, Poland; 2 The School of Medicine, Medical Sciences and Nutrition, University of Aberdeen, Aberdeen AB24 3FX, UK; 3 Research Centre for Health, Glasgow Caledonian University, Glasgow G4 0BA, UK

**Keywords:** megaprosthesis, orthopaedic oncology, arthroplasty, rehabilitative outcomes

## Abstract

Background/Objectives: Bone reconstruction using megaprostheses is increasingly performed following bone tumour resections, including sarcomas, to enhance patient outcomes and quality of life. However, this is a complex patient group, and there is little consensus as to postoperative rehabilitation and associated outcomes. Methods: A systematic search was conducted in MEDLINE and EMBASE databases according to the Implementing Prisma in Exercise, Rehabilitation, Sport medicine and SporTs science (PERSiST) guidelines. Studies describing rehabilitation protocols and functional outcomes following bone tumour resection and modular oncologic megaprosthesis reconstruction were included. All papers were individually assessed for methodological quality using the Joanna Briggs Institute (JBI) critical appraisal tool. Results: The search generated 105 records, 28 underwent full-text review, and 13 studies were included. Available data reflect 371 patients with a mean age of 49.17 (S.D. 21.40) years and a mean postoperative follow-up of 41.88 (S.D. 32.88) months. Surgical indications were documented as sarcomas in 9 studies, and tumour metastasis to the bone in 10 studies. Rehabilitation protocols were reported in 5 studies following proximal humerus resection with a mean dislocation rate of 14.5% (S.D. 5.26). All protocols advised brace immobilisation for a period ranging between 10 days and 6 months. Superior Constant-Murley shoulder score was reported in patients with early active isometric exercises at 6 weeks. Six studies reported proximal femur prosthesis rehabilitation and functional outcomes, with a mean dislocation rate of 10% (S.D. 9.82). Enhanced outcomes were reported in studies employing early mobilisation. Two studies assessed distal femur prosthesis; both studies reported similar protocols with full weight bearing 3 weeks following surgery. The methodological quality of the studies varied, but was overall modest, with 10/13 studies meeting at least 50% of JBI reporting criteria. Conclusions: The existing literature on rehabilitation and outcomes in orthopaedic oncology patients following arthroplasty with megaprosthesis is limited, with rehabilitative protocols variably described. However, it seems that early active mobilisation does not increase the risk of joint dislocations or infections.

## Simple Summary

When bone cancer, such as sarcoma, requires the removal of a large section of bone, surgeons can replace the missing bone with a megaprosthesis—a large implant designed to fill the gap and restore limb function. These complex surgeries can help patients regain movement and improve quality of life, but there is little agreement on the best recovery approach afterwards. We reviewed studies describing rehabilitation programmes and patient outcomes following megaprosthesis surgery. Recovery strategies varied greatly, with some encouraging earlier movement and others using longer immobilisation. Evidence suggests that starting gentle movement earlier may improve function without increasing the risk of complications such as joint dislocation or infection. However, research in this area is still limited, and more high-quality studies are needed to determine the most effective rehabilitation plans for patients undergoing this type of reconstruction.

## 1. Introduction

Bone tumours present a significant challenge in orthopaedic oncology, often necessitating extensive surgical interventions. Improved life expectancy of cancer patients combined with earlier diagnosis of primary sarcomas has resulted in an increased prevalence of detected bone lesions [1,2]. As a result, increasing numbers of bone tumour resections with megaprosthesis reconstruction have been performed, which has facilitated improved patient morbidity and mortality outcomes as well as an increase in patient quality of life in the palliative setting [3]. Rehabilitation following such significant surgery in complex patients is challenging. Although the literature on clinical outcomes following arthroplasty with megaprostheses in orthopaedic oncology has increased in recent years, there is little consideration given to the rehabilitation patients receive. There are no specific rehabilitation guidelines to guide clinical management of this complex and diverse group.

During the last few decades, the use of modular megaprostheses such as the Modular Universal Tumour and Revision System (MUTARS) has revolutionised the surgical management of patients with malignant bone tumours [4]. Megaprosthesis indications include reconstruction after resection of primary malignant bone tumours, as well as in cases of locally aggressive benign tumours of the bone. Occasionally, malignant soft-tissue sarcomas that are in proximity to, or engage, the bone sometimes require skeletal reconstruction if a wide surgical margin is to be achieved. Equally, megaprosthesis reconstruction may be performed in patients with pathological fractures or impending fractures in those with an underlying oncological condition. Immediate weight bearing is allowed post-arthroplasty, which is a strong argument for its use, as it bypasses the need for fracture healing and optimises quality of life, even in patients with unfavourable prognoses [5]. However, these complex procedures can lead to considerable changes in musculoskeletal function. Modular megaprosthesis is associated with greater soft tissue injury and muscle dysfunction compared with standard prosthetics used in routine arthroplasty for osteoarthritis. It is reported that the rates of dislocations and local soft tissue complications are far greater in megaprosthesis, necessitating effective rehabilitation protocols to optimise patient outcomes [6]. Previous megaprosthesis reviews have assessed outcomes in non-oncologic settings [7]. However, trauma and revision arthroplasty patients are a different group, as although possibly frail in presentation, they are unaffected by cancer cachexia, which has profound implications for postoperative outcomes, directly associated with increased rates of complications following surgery [8].

Despite the importance of rehabilitation in the recovery process, there is a lack of consensus regarding the most effective rehabilitation protocols and their impact on functional outcomes for patients undergoing bone tumour resection and prosthetic reconstruction. This systematic review aimed to evaluate published rehabilitation protocols and their suggested impact on functional outcomes following bone tumour resection and modular megaprosthesis reconstruction.

## 2. Materials and Methods

A systematic review was conducted according to the updated Preferred Reporting Items for Systematic reviews and Meta-Analyses (PRISMA) guidelines [9] (Supplementary Materials). The search was performed in Ovid MEDLINE and EMBASE databases for English-language published papers from inception until 22 December 2024. Grey literature searches were performed for the same timescale. The search was conducted by a single author (MB), and two authors (MB and FB) independently screened titles and abstracts. In cases of disagreements, papers were included for full review. This systematic review was not prospectively registered in PROSPERO or any other database.

The applied search terms in databases included: “rehabilitation” [MeSH terms] OR “recovery” [MeSH terms] OR “rehabilitation protocol” [MeSH terms] OR “postoperative protocol” [MeSH terms] AND “MUTARS” [MeSH terms] OR “megaprosthesis” [MeSH terms] OR “Modular Universal Tumour and Revision System” [MeSH terms] OR “Resectional Prosthesis” OR “Tumour and Revision Prosthesis”. Independent searches using the same MeSH terms were carried out in each database, and the results were combined.

Studies describing rehabilitation protocols and functional outcomes following primary sarcoma and metastasis bone resection treated with modular megaprosthesis reconstruction were included. Only studies in an oncologic setting were included. Studies where operative indications were related to trauma and prosthesis revision surgery were excluded. All oncologic modular megaprosthesis reconstructions were considered except pelvic reconstructions. Due to the nature of the information sought, controlled trials, cohort studies, case-control studies and case-series were considered. Non-clinical studies, reviews, individual case reports, and conference abstracts were excluded. Studies were also excluded where rehabilitation protocols and functional outcomes were not clearly defined or reported. Studies that described mixed cohorts of megaprostheses surgery and outcomes (such as hip and shoulder procedures) were included where joint-specific outcome distinction was made. Studies that described mixed cohorts but with a single mean outcome and no distinction as to joint-specific results were excluded.

One author (MB) performed the data extraction into a bespoke data capture spreadsheet. Relevant data included: authors, study date, study type, number of participants, patient demographics, type of modular megaprosthesis and reconstruction performed. The primary data sought for this review were the rehabilitative protocol parameters and functional outcomes. We also extracted mortality rates, postoperative complications and data about study methodology as secondary measured variables.

All papers were individually assessed for methodological quality by 2 authors (MB and FB) using the Joanna Briggs Institute (JBI) critical appraisal tool for case series [10]. The tool comprises a 10-point checklist addressing study design and reporting, with the following: Yes, No, Unclear and Not applicable selection options for each component.

## 3. Results

### 3.1. Study Characteristics

**Figure 1:**
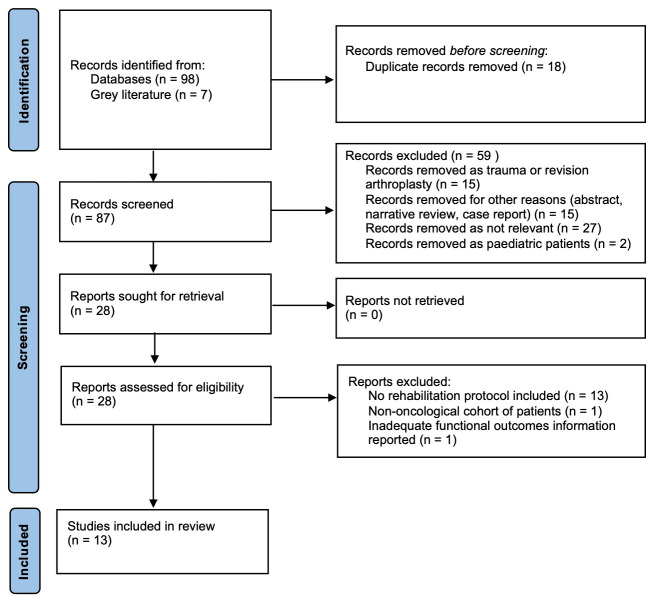
2020 Prisma flow diagram showing the systematic selection of records.

The literature search generated 98 records. An additional seven records were added from the grey literature. Following the removal of duplicates, 87 papers were evaluated against the eligibility criteria. After screening, 28 were eligible for full-text review. In total, 13 studies were included in the final data analysis (Figure 1). All included studies were case series, with 12 of the 13 studies having retrospective data collection. In total, data were available for 371 patients; the female ratio was 140:146 as reported in 10 of the 13 studies. The mean age of participants in the studies was 49.17 (S.D. 21.40) years.

### 3.2. Performed Operations, Morbidity and Mortality Outcomes

Across the 13 papers reporting tumour resection and megaprosthesis reconstruction, 5 studies reported a rehabilitation protocol and assessed functional outcomes following proximal humerus, 1 study following distal humerus, 2 studies following total femur, 6 studies following proximal femur, 2 studies following distal femur and 1 study following proximal tibia. Of note, Shehadeh et al. reported rehabilitation protocols and outcomes for multiple joints [11]. Surgical indications were documented as primary sarcomas in 9 studies, and tumour metastasis to the bone in 10 studies. The mean patient follow-up period where final functional assessment was performed was 41.88 (S.D. 32.88) months. Mortality was assessed in 9/13 studies, with a mean mortality rate of 18.6% (S.D. 21.37) at a mean time point of 44.57 months (S.D. 34.54). Postoperative surgical site and deep prosthesis infection rate was recorded in 9/13 studies with an average rate of 15.4% (S.D. 17.09) (Table 1).

### 3.3. Rehabilitative Protocols and Functional Outcomes

#### 3.3.1. Shoulder Reconstruction

**Figure 2:**
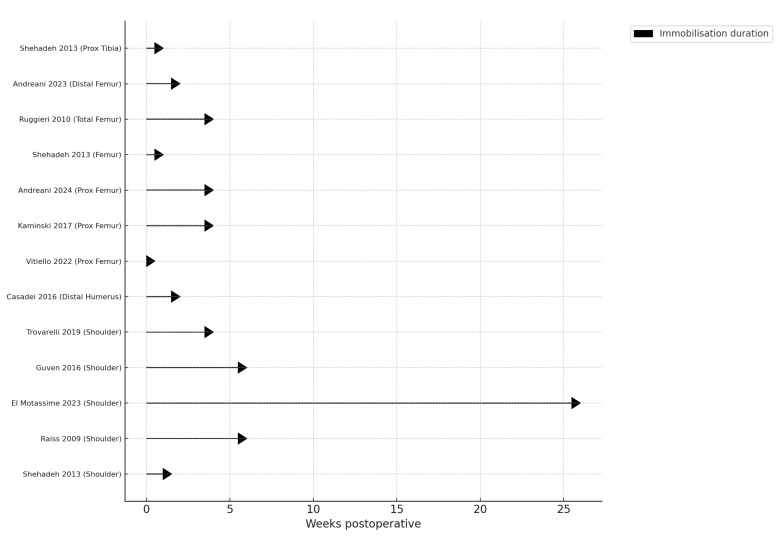
Reported immobilisation duration across included studies, expressed in weeks. Each arrow represents the immobilisation period before initiation of rehabilitation.

In the 5/13 studies documenting proximal humerus resections, rehabilitation protocols utilised either brace or sling immobilisation, with the range of immobilisation period ranging from 10 days to 6 months [11,13]. Only 1 study utilised a 30-degree abduction brace. Troverelli et al. reported a superior Constant shoulder score in patients who were instructed to perform early active isometric exercises at 6 weeks, in contrast to Guven et al.; however, this may be a result of a longer follow-up period (36 vs. 18.2 months) (Figure 2). The rate of prosthesis dislocations was reported in 4/5 studies with a mean rate of 14.5% (S.D. 5.26) (Table 2). Casadei et al. was the only study to assess distal humerus resections (Table 3). Similarly, the arm was immobilised in a sling for 2 weeks. The duration of arm immobilisation was dependent on whether a cemented or uncemented prosthesis was employed. There was a disparity in outcomes noted for primary tumour or a metastatic lesion, with mean elbow ROM after resection being 70◦ in patients with primary tumour and 40° in those with metastasis. Mean Mayo Elbow Performance (MEP) and the Musculoskeletal Tumour Society Score (MSTS) scores were respectively 84% and 22/30 (73%).

#### 3.3.2. Hip Reconstruction

Proximal femur or total femur prosthesis was reported in 7/13 studies. Proximal femur prosthesis rehabilitation protocol and functional outcomes were discussed in six of these. Vitiello et al. reported a score of 0.5 (measured on a scale of 1 to 10) on the Visual Analogue Scale (VAS) at 12 months, which was notably lower than the 3.4 points reported by Kamiński [17,18]. Vitello et al. advised earlier mobilisation without crutches or walking frames, with patients all able to weight bear independently at 2 months [17]. In contrast, Kamiński et al. advised full weight bearing by the end of the 4th month [18]. Vitello et al. reported a MSTS score of 19.1 at 12 months, whereas Andreani et al. reported a MSTS score of 23.2 at 44.2 months [19]. Andreani et al., however, suggest a more conservative rehabilitative protocol, advising the use of a hip brace for up to 4 weeks following surgery [19]. Shehadeh et al. and Pitera et al. utilised the same rehabilitation protocol, with the former study reporting a MSTS score of 86% between 4- and 8-months after surgery, and the latter a score of 66% at 6 weeks following surgery [11,22]. Prosthesis dislocation rates were reported in 5/6 studies assessing proximal femur prosthesis with a mean rate of 10% (S.D. 9.82). Outcomes for total femur prosthesis were discussed in 2/13 studies. Ruggieri et al. suggested a strict immobilisation in a cast regime for the first 4 weeks compared to Shehadeh et al., suggesting mobileisation in a custom brace following surgery instead (Figure 2). Shehadeh et al. report a higher MSTS score of 86% between 4- and 8-months compared to Ruggieriet et al., who report a score of 66% after 6 months of rehabilitation (Table 4).

#### 3.3.3. Knee Reconstruction

Overall, 5/13 studies involved tumour resections, which required resection and arthroplasty of the knee joint. Two of those studies assessed distal femur prosthesis, one assessed proximal tibia prosthesis and two assessed total femur rehabilitation protocols. For distal femur resections, both studies had similar rehabilitative protocols with full weight bearing around the 3rd week following surgery. Shehadeh et al., however, made a distinction in rehabilitative protocol with early weight bearing as tolerated following the 3rd day after surgery for cemented prostheses and partial weight bearing for cementless prostheses [11]. Similarly, only Shehadeh et al. described a rehabilitative protocol for proximal tibia resections with immediate early weight bearing as tolerated following surgery and knee flexion exercises following a 6-week period after surgery. Functional outcome measures varied between studies (Table 5).

### 3.4. Study Quality Assessment

The methodological quality of the studies varied considerably (Table 6). 8/13 studies met at least 6 of the 10 evaluation criteria, and a further 2 (10/13) met at least 50% of items. 3 studies failed to reach 50% of the reporting criteria.

## 4. Discussion

This is the first review to specifically consider the rehabilitation processes followed in patients who underwent arthroplasty with modular megaprostheses in orthopaedic oncology and consider the influence on functional outcomes. Despite heterogeneity in the outcome measures reported and no comparative trials contrasting approaches, the results broadly suggest that early active mobilisation of both upper and lower limbs following surgery, and early weight bearing following lower limb reconstruction, perhaps offer more favourable functional outcomes.

We found comparatively few published studies (n = 13) that report postoperative rehabilitative protocols and outcomes for patients with an active malignancy, and the cancer cachexia that is likely associated. In particular, cancer cachexia contributes to muscle wasting, impaired immune function, and delayed wound healing, all of which may negatively affect rehabilitation capacity. These pathophysiological differences distinguish oncological patients from trauma or revision cohorts and must be considered when interpreting functional outcomes [24]. We therefore excluded another 16 studies which reported outcomes for modular megaprosthesis in trauma and revision arthroplasty cohorts. Trauma and revision arthroplasty patients are likely a different group, as although possibly frail in presentation, they are unaffected by cachexia, which is characterised by severe weight loss, muscle wasting, and has profound implications for postoperative outcomes in surgical patients; being directly associated with increased morbidity, prolonged hospital stays, and higher rates of complications following surgery [8]. Further, patients exhibiting cachexia often experience impaired immune function, delayed wound healing, and reduced muscle strength, which can hinder recovery and rehabilitation efforts. Because of this, it was essential to make a distinction between trauma patients and cancer patients in the context of limb reconstruction, as the underlying mechanisms and clinical implications of cachexia may differ significantly between these groups. Future rehabilitative programs following modular megaprosthesis should take into consideration the metabolic burden of cancer cachexia in this group of patients, allowing for tailored preoperative and postoperative management strategies. Moreover, the use of preoperative cancer cachexia scores such as the cachexia index may aid with preoperative planning as well as determining the patient’s rehabilitative potential following surgery, mitigating the metabolic effect of sarcopenia and enhancing recovery and outcomes [25].

In upper limb reconstruction and specifically in the proximal humerus resections, all reported rehabilitation protocols utilised brace or sling immobilisation, with the range of immobilisation period varying widely, from 10 days to 6 months [11,13]. Troverelli et al. reported a superior Constant-Murley shoulder score in patients with early active isometric exercises at 6 weeks compared to Guven et al. [14,15]. Previous meta-analysis of outcomes in patients following shoulder modular megaprosthesis (with trauma as well as oncological-related indications), however, reported a moderately higher mean Constant-Murley Score of 63 compared with the studies included in this review. These findings, however, may be a result of differences in outcomes for patients with oncological and trauma-related indications [26]. Casadei et al. similarly reported poorer functional outcomes and ROM in patients with metastatic disease compared with primary tumour resection of the distal humerus, which may be a result of cachexia-driven muscle decline [16]. In distal humerus resections, the limb was similarly immobilised in a sling in the initial postoperative period. Active elbow movement was dependent on whether the prosthesis was fixed using a press-fit method or using cement. Trung et al. report beginning active elbow motion on the 10th day following surgery and strength training 14 days after surgery using cement to fix the prosthesis. The reported functional outcomes 15 months after surgery were 140 degrees of active flexion, 0 degrees of active extension, 90 degrees of active pronation, and 80 degrees of active supination. In this series, Mayo’s function score reached an excellent level at 100 points [27].

In lower limb reconstructions, rehabilitation with earlier fully weight-bearing mobilisation was associated with lower pain on the VAS score (0.5 following earlier mobilisation and 3.4 with prolonged immobilisation) [17,18]. MSTS scores were also higher in studies advising earlier mobilisation without brace use [23]. However, rates of hip dislocations were not reported in all included studies and whether more aggressive mobilisation without the use of hip braces is associated with increased subluxation risk is unknown. Based on the included studies, early weight bearing did not appear to increase the risk of prosthesis dislocation. Previous authors have suggested the rate of hip dislocations in this population is 14.6% [28]. Kamiński et al. reported 15.3% (2/13) in their group of cancer patients; however, Vitiello et al. reported a 4% rate of prosthesis dislocation despite a more aggressive rehabilitative protocol [17]. However, due to heterogeneity and inconsistent reporting, this finding should be interpreted cautiously.

This study has several limitations. The studies included were heterogeneous, with varying anatomical locations of bone resection. Further, included studies contained a variety of functional outcome measures, which were not directly comparable, effectively inhibiting data synthesis. The operative techniques employed may have varied between cohorts and surgeons, and may have been contributory variables affecting outcomes. The degree of bone resection due to tumour size may have varied between individual cases and studies, contributing to the variation in local trauma to soft tissues and the size of the inserted modular megaprosthesis. Similarly, there was likely variation in the degree of soft tissue resection due to local tumour spread to surrounding connective tissues, influencing postoperative rehabilitation requirements and outcomes. Soft tissue reconstruction and muscle flap reattachment may be aided by attachment of a tube mesh; however, this is not always performed [29]. Another important limitation is the lack of data on surgeon expertise or surgical learning curves. Variation in surgical technique and skill level may have influenced complication and functional outcomes, but the included studies did not provide sufficient detail to assess this factor. Despite several papers reporting outcomes following modular megaprosthesis reconstruction, overall, the literature describing rehabilitative protocols is limited. In several papers, rehabilitation protocols are not well described, which has contributed to the overall modest JBI scores relating to the methodological quality of included studies.

## 5. Conclusions

The existing literature on rehabilitation and outcomes in orthopaedic oncology patients following arthroplasty with modular prosthesis is limited, with rehabilitative protocols variably described. However, it seems that early active mobilisation does not increase the risk of joint dislocations or infections and likely leads to enhanced quality of life. Future prospective studies are needed to directly compare early versus delayed mobilisation protocols and to develop standardised rehabilitation pathways tailored for oncological patients.

## Figures and Tables

**Table 1:** Study characteristics data.

Author Year Country	Study	Surgical Indication	Number of Participants (Male: Female Ratio) Mean Age (Years)	Follow-Up Period (Months)	Mortality Outcomes Morbidity Outcomes Other Outcomes
Proximal humerus					
Shehadeh et al. 2013 [11] Jordan	Retrospective	Primary bone tumour	59 (32:27) * 11 patients eligible 24	24	No mortality data No infection rate data
Raiss et al. 2009 [12] Germany	Prospective	Primary tumour and metastatic disease	39 (19:20) 60	38	Mortality rate was 10% at 11.5 years 5% of patients had deep wound infections 10% prosthesis dislocation rate
El Motassime et al. 2023 [13] Italy	Retrospective	Metastatic disease	20 (12:8) 61.5	21	17.9% mortality rate at 6 months 10% prosthesis dislocation rate No infection data reported.
Guven et al. 2016 [14] Turkey	Retrospective	Primary bone tumour and metastatic disease	10 (5:5) 49.4	18.2	20% mortality rate at 6 months No infections recorded 20% prosthesis dislocation rate
Trovarelli et al. 2019 [15] Italy	Retrospective	Primary tumour	22 (9:13) 55	36	22.7% mortality rate at 22 months Aseptic loosening in 4% of patients No infections recorded. 18% prosthesis dislocation rate
Distal Humerus					
Casadei et al. 2016 [16] Italy	Retrospective	Primary bone tumour and metastatic disease	47	35	31.9% mortality rate at 35 months 4% wound infection rate
Proximal and Total Femur					
Shehadeh et al. 2013 [11] Jordan	Retrospective	* Total femur and proximal femur Primary bone tumour.	59(32:27) * 6 patients eligible 24	24	No available mortality data. No infection rate data.
Vitiello et al. 2022 [17] Italy	Retrospectively	Proximal femur Metastatic disease	25 (10:15) 67.5	30.5	No available mortality data. No infections noted. Other infections: cystitis 8%, pneumonia 8%. 4% prosthesis dislocation rate
Kamiński et al. 2017 [18] Poland	Retrospective	Proximal femur Metastatic disease and implant revision	34 (7:6) 68.5	18	11.7% mortality rate at 12 months 0 reported surgical site infections 15% prosthesis dislocation rate
Andreani et al. 2024 [19] Italy	Retrospective	Proximal femur Primary bone tumour and metastatic disease	22 (missing ratio) 58.9	44	No available mortality data. 4% superficial wound infection rate 4% prosthesis dislocation rate
Ziranu et al. 2022 [20] Italy	Retrospective	Proximal femur Metastatic disease	35 (12:23) 72	34.5	4% mortality rate at 2 years 50% periprosthetic joint infection 25% prosthesis dislocations and tendon ruptures
Ruggieri et al. 2010 [21] Italy	Retrospective	Total femur Primary bone tumour	21 (15:6) 21	120	55% mortality rate at 10 years 14% periprosthetic joint infection 4% prosthesis dislocations
Pitera et al. 2017 [22] Poland	Retrospective	Proximal femur Primary bone tumour and metastatic disease	42 (19:23) 63	1.5	No available mortality data No available wound infection rate 2% prosthesis dislocation rate
Distal Femur and Proximal Tibia					
Shehadeh et al. 2013 [11] Jordan	Retrospective	Proximal tibia Primary bone tumour	59(32:27) * 8 patients eligible 24	24	No mortality data. 37.5% infection rate
Shehadeh et al. 2013 [11] Jordan	Retrospective	Distal femur Primary bone tumour	59(32:27) * 21 patients eligible 24	24	No mortality data. No infection rate data.
Andreani et al. 2023 [23] Italy	Retrospective	Distal Femur Primary bone tumour and metastatic disease	16 44.1	46.7	No mortality data. 6.25% wound infection rate

* *Number of patients considered in the study who underwent megaprosthesis reconstruction due to oncological indications.*

**Table 2:** Proximal humerus prosthesis rehabilitation protocols and functional outcomes.

Study	Rehabilitation Protocol	Functional Outcomes
Shehadeh et al. 2013 [11] Jordan	Days 1–10: Arm in sling (or immobiliser). Start hand and elbow exercises. Day 10: Take off arm sling for gentle Codman I/II shoulder exercises. Start elbow full extension exercise after week 4. 6 weeks of AAROM shoulder.	MSTS–ISOLS Score: 83%
Raiss et al. 2009 [12] Germany	Day 1: Arms placed in internal rotation in Gilchrist bandages for 6 weeks, shoulder mobilised passively for 6 weeks with 60 degrees of shoulder flexion and abduction and 0 degrees of external rotation 6 weeks: unlimited range of motion exercises.	Mean Enneking score: 19 (range: 7–27 points) at last follow-up (38 months) Mean active ROM: shoulder flexion 34° (range: 0–90°), abduction 33° (range: 0–90°), and external rotation 10° (range: 10–50°).
El Motassime et al. 2023 [13] Italy	Day 1: Brace immobilisation Day 15: Codman exercises and elbow flexion-extension exercises 6 months: brace removal, active ROM exercises.	Mean MSTS score: 57.6% (±26.24) Mean DASH score: 47.5 (±27.55) Mean WOSI score: 950 (58.62%) (±532.29)
Guven et al. 2016 [14] Turkey	Day 1: Sling immobilisation with an abduction pillow. Passive wrist and elbow exercises. Week 6: Passive shoulder exercises	Mean active flexion: 96° (range, 30–160°), mean active abduction: 88 (range: 30–160°), mean active external rotation: 13° (range: 0–20°). Mean Constant-Murley score: 53.7% (range: 22–96%) Mean DASH score: 26.2 (range: 5.8–60) Mean VAS score: 1.3 (range: 0–4) Mean MSTS score: 78.1% (range: 50–93%)
Trovarelli et al. 2019 [15] Italy	Day 1: Postoperative 30° abduction brace for 4 weeks. 1 month: active mobilisation with pendulum movements limited to 30° of abduction, forward flexion and extension Week 6–7: isometric exercise to emphasise lower trapezius and serratus anterior activation and reduce upper trapezius activation.	Mean Constant score: 61 (42 to 89), mean normalised Constant score: 66 (48 to 97) Mean ASES score: 81 (range: 62 to 92) Mean MSTS score was 29% (range: 26 to 30) Mean abduction: 103° (range: 40 to 180°), mean flexion: 117° (range: 40 to 180°) and mean external rotation: 58° (range: 45 to 75°)

Where: MSTS–ISOLS: Musculoskeletal Tumour Society–International Symposium on Limb Salvage, MSTS: The Musculoskeletal Tumour Society rating system, DASH: Disability of Arm- Shoulder-Hand, WOSI: Western Ontario Shoulder Instability Index, ASES: American Shoulder and Elbow Surgeons Score, AAROM: active-assisted range of motion, ROM: range of motion, VAS: visual analogue scale.

**Table 3:** Distal humerus prosthesis rehabilitation protocols and functional outcomes.

Study	Rehabilitation Protocol	Functional Outcomes
Casadei et al. 2016 [16] Italy	0–2 weeks: Arm immobilised in sling. Active and passive finger movement was initiated on the first postoperative day. Week 1: Active elbow movement in cemented prostheses, Week 4: Active arm movements in uncemented prostheses, Week 6–8: Active arm movement in allograft-prosthesis composite	Mean elbow ROM: 70° in patients with primary tumour, 40° in patients with metastasis. Mean MEP score: 84% Mean MSTS score 73%

Where: MSTS: The Musculoskeletal Tumour Society rating system, MEP: Mayo Elbow Performance.

**Table 4:** Proximal and total femur prosthesis rehabilitation protocols and functional outcomes.

Study	Rehabilitation Protocol	Functional Outcomes
Vitiello et al. 2022 [17] Rome	Day 2: patients seated with their feet out of bed. Day 3: progressive weight bearing with walker frames. Week 8: All patients walking without aids.	Mean Karnofsky score: 76% (±21) Mean VAS at 1 month, 6 months and 12 months—2.1, 0.5 ± 1.2 and 0.5 ± 0.8, respectively. Mean MSTS at 1 month, 6 months and 12 months—12.3 ± 3.7, 19.2 ± 2.4 and 19.1 ± 5.6, respectively.
Shehadeh et al. 2013 [11] Jordan	Days 1–3: Limb suspended in abduction (30°) and flexion (30°). Knee and ankle exercises. For the total femur, in addition, the knee is immobilised in knee brace. Day 4: Week 6: The patient is mobilised in a custom abduction brace (locked in 30° abduction and 0–60° hip flexion), toe touch weight bearing started. Abductor muscles strengthening. For the total femur, the knee immobiliser was discontinued at two weeks, and knee flexion exercises started. Week >6–8: Brace is removed (Active hip abduction required before the brace is removed, and full weight bearing is allowed)	Mean MSTS–ISOLS score: 86%
Kamiński et al. 2017 [18] Poland	Week 0–6: Walking with crutches and partial load. This period was extended to 12 weeks in cases where concomitant acetabular reconstruction prevented the patients from earlier weight bearing. Week >6: Gradually achieve full load on the limb (no later than 4 months after surgery).	Mean VAS score: 3.4 Mean HHS: 70.68 Mean modified Harris Hip Score: 64.25
Andreani et al. 2024 [19] Italy	Week 1–2: Hip brace 0–30° for full time. Toe touch weight bearing on the operated leg Week 2–3: Hip brace 0–30° for full time. Partial weight bearing on the operated leg Week 3–4: Hip brace 0–60° for full time. Progressive partial weight bearing on the operated leg Week >4: Hip brace 0–60° (up to 0–90° in selected cases) for full-time for at least 2 months and progressive removal. Full weight bearing on the operated leg. Stair climbing re-education is most intense	Mean MSTS score: 23.2
Ziranu et al. 2022 [20] Italy	Day 2: patients seated with their feet out of bed Day 3: Progressive weight bearing with walker frames. Routine total hip precautions were followed for 3 months.	Walking without aids was achieved in 2 months for all patients.
Ruggieri et al. 2010 [21] Italy	Day 1: Isometric exercises were started the day after surgery. Week 1–4: Immobilisation in a cast. Assisted walking for 6 weeks, supervised by a physical therapist. Week 8: Brace with progressive ROM for a further 2 months.	Mean MSTS: 66% Average knee ROM: 60° (range: 0–110°)
Pitera et al. 2017 [22] Poland	Days 1–3: The limb is suspended in abduction (30°) and flexion (30°). Knee and ankle exercises are encouraged. For the total femur, in addition, the knee is immobilised in a knee brace. Day 4—week 6—The patient is mobilised in a custom abduction brace (locked in 30° abduction and 0–60° hip flexion), toe touch weight bearing started. Abductor muscles strengthening. For the total femur, the knee immobiliser was discontinued at two weeks and knee flexion exercises start. Week 6–8: Brace is removed. Active hip abduction is required before the brace is removed, and full weight bearing is allowed.	Mean VAS score: 3.8 Mean MSTS score: 66% Mean HHS score: 75

Where: VAS: Visual Analogue Scale Score, MSTS: The Musculoskeletal Tumour Society rating system, HHS: Harris Hip Score.

**Table 5:** Distal femur and proximal tibia prosthesis rehabilitation protocols and functional outcomes.

Study	Rehabilitation Protocol	Functional Outcomes
Shehadeh et al. 2013 [11] Jordan	Distal Femur: Day 1–3: knee immobiliser, start isometric exercises, knee flexion NOT allowed. Bed to chair only. Day 3 to week 2: Start weight bearing as tolerated for cemented prostheses (with knee immobiliser). For cementless prostheses, partial weight bearing (with knee immobiliser). Week 2–6: AAROM knee if skin healed. Discontinue the knee brace. Continue concentration on extensor strengthening. Begin hamstring exercises. Week > 6: Knee flexion exercises and increase the extensor strength. Proximal Tibia: Day 1–5: Rigid knee immobiliser. Allow weight bearing as tolerated. Day 5 to week 6: No active or passive knee flexion. Keep the knee in an immobiliser to allow healing of the patellar tendon. Isometric quadriceps strengthening exercises only. >6 weeks: passive and gentle AAROM knee flexion.	Mean MSTS–ISOLS score: 93% in the distal femur patient cohort Mean MSTS–ISOLS score: 88% in proximal tibia patient cohort
Andreani et al. 2023 [23] Italy	Week 1–2: Knee brace. Partial weight bearing. Gait re-education, postural passages. Week: 2–3: Progressive weight bearing. Week: 3–4: Full weight bearing. Progressive stair climbing. Week >4: High-intensity stair climbing exercises. Removal of brace at day 30.	Mean MSTS score: 23.2 (range: 12–30)

Where: MSTS: The Musculoskeletal Tumour Society rating system, MSTS–ISOLS: Musculoskeletal Tumour Society–International Symposium on Limb Salvage.

**Table 6:** Methodological quality assessment using the Joanna Briggs Institute (JBI) critical appraisal tool.

Study	Andreani et al. 2024 [19]	Andreani et al. 2023 [23]	El Motassime et al. 2023 [13]	Vitiello et al. 2022 [17]	Ziranu et al. 2022 [20]	Trovarelli et al. 2019 [15]	Kamiński et al. 2017 [18]	Pitera et al. 2017 [22]	Casadei et al. 2016 [16]	Guven et al. 2016 [14]	Shehadeh et al., 2013 [11]	Ruggieri et al. 2010 [21]	Raiss et al. 2009 [12]
Were there clear criteria for inclusion in the study?	YES	YES	UNCLEAR	UNCLEAR	NO	YES	UNCLEAR	YES	YES	YES	NO	YES	YES
Was the condition measured in a standard, reliable way for all participants included in the case series?	YES	YES	NO	NO	NO	NO	NO	YES	NO	NO	NO	YES	YES
Were valid methods used for the identification of the condition for all participants included in the case series?	YES	YES	NO	NO	YES	YES	NO	NO	YES	YES	NO	YES	NO
Did the case series have consecutive inclusion of participants?	YES	YES	YES	YES	NO	YES	UNCLEAR	UNCLEAR	YES	YES	YES	YES	YES
Did the case series have complete inclusion of participants?	YES	YES	NO	YES	YES	NO	NO	UNCLEAR	NO	NO	UNCLEAR	NO	NO
Was there clear reporting of the demographics of the participants in the study?	NO	NO	YES	YES	YES	YES	YES	YES	NO	YES	YES	YES	YES
Was there clear reporting of clinical information of the participants?	YES	YES	YES	YES	YES	YES	YES	YES	YES	YES	YES	YES	YES
Were the outcomes or follow-up results of cases clearly reported?	NO	NO	YES	YES	NO	YES	YES	YES	YES	YES	YES	YES	YES
Was there clear reporting of the presenting site(s)/clinic(s) demographic information?	YES	YES	YES	YES	UNCLEAR	YES	YES	YES	UNCLEAR	YES	UNCLEAR	YES	YES
Was statistical analysis appropriate?	YES	YES	NO	YES	NO	NO	NO	NO	NO	NO	NO	NO	NO

## Supplementary Materials

The following supporting information can be downloaded at: https://www.mdpi.com/article/10.3390/cancers17182951/s1.

## References

[1] Bartelstein M.K., Boland P.J. (2022). Fifty Years of Bone Tumors. J. Surg. Oncol..

[2] Stomeo D., Tulli A., Ziranu A., Perisano C., De Santis V., Maccauro G. (2015). Acrometastasis: A Literature Review. Eur. Rev. Med. Pharmacol. Sci..

[3] Thorkildsen J., Strøm T.A., Strøm N.J., Sellevold S., Norum O.-J. (2022). Megaprosthesis for Metastatic Bone Disease—A Comparative Analysis. Curr. Oncol..

[4] Standard/Tumour Prothetics-Implantcast. https://www.implantcast.de/en/for-medical-professionals/products/standard-/-tumour-prosthetics/.

[5] Gkavardina A., Tsagozis P. (2014). The Use of Megaprostheses for Reconstruction of Large Skeletal Defects in the Extremities: A Critical Review. Open Orthop. J..

[6] Puchner S.E., Funovics P.T., Hipfl C., Dominkus M., Windhager R., Hofstaetter J.G. (2014). Incidence and Management of Hip Dislocation in Tumour Patients with a Modular Prosthesis of the Proximal Femur. Int. Orthop..

[7] Sambri A., Parisi S.C., Zunarelli R., Di Prinzio L., Morante L., Lonardo G., Bortoli M., Montanari A., De Cristofaro R., Fiore M. (2023). Megaprosthesis in Non-Oncologic Settings-A Systematic Review of the Literature. J. Clin. Med..

[8] Nishikawa H., Goto M., Fukunishi S., Asai A., Nishiguchi S., Higuchi K. (2021). Cancer Cachexia: Its Mechanism and Clinical Significance. Int. J. Mol. Sci..

[9] Page M.J., McKenzie J.E., Bossuyt P.M., Boutron I., Hoffmann T.C., Mulrow C.D., Shamseer L., Tetzlaff J.M., Akl E.A., Brennan S.E. (2021). The PRISMA 2020 Statement: An Updated Guideline for Reporting Systematic Reviews. BMJ.

[10] JBI Critical Appraisal Tools|JBI. https://jbi.global/critical-appraisal-tools.

[11] Shehadeh A., Dahleh M.E., Salem A., Sarhan Y., Sultan I., Henshaw R.M., Aboulafia A.J. (2013). Standardization of Rehabilitation after Limb Salvage Surgery for Sarcomas Improves Patients’ Outcome. Hematol. Oncol. Stem Cell Ther..

[12] Raiss P., Kinkel S., Sauter U., Bruckner T., Lehner B. (2010). Replacement of the Proximal Humerus with MUTARS Tumor Endoprostheses. Eur. J. Surg. Oncol..

[13] El Motassime A., Meschini C., Di Costa D., Rovere G., Matrangolo M.R., De Maio F., Farsetti P., Ziranu A., Maccauro G., Vitiello R. (2023). Functional Outcomes and Shoulder Instability in Reconstruction of Proximal Humerus Metastases. Curr. Oncol..

[14] Guven M.F., Aslan L., Botanlioglu H., Kaynak G., Kesmezacar H., Babacan M. (2016). Functional Outcome of Reverse Shoulder Tumor Prosthesis in the Treatment of Proximal Humerus Tumors. J. Shoulder Elbow Surg..

[15] Trovarelli G., Cappellari A., Angelini A., Pala E., Ruggieri P. (2019). What Is the Survival and Function of Modular Reverse Total Shoulder Prostheses in Patients Undergoing Tumor Resections in Whom an Innervated Deltoid Muscle Can Be Preserved?. Clin. Orthop..

[16] Casadei R., De Paolis M., Drago G., Romagnoli C., Donati D. (2016). Total Elbow Arthroplasty for Primary and Metastatic Tumor. Orthop. Traumatol. Surg. Res. OTSR.

[17] Vitiello R., Perisano C., Greco T., Cianni L., Polichetti C., Comodo R.M., De Martino I., La Vergata V., Maccauro G. (2022). Intramedullary Nailing vs Modular Megaprosthesis in Extracapsular Metastases of Proximal Femur: Clinical Outcomes and Complication in a Retrospective Study. BMC Musculoskelet. Disord..

[18] Kamiński P., Szmyd J., Ambroży J., Jaworski J.M., Frańczuk B. (2017). A Comparison of Outcomes of Treatment with Resection Prosthesis of the Hip in Revision and Oncological Surgery. Ortop. Traumatol. Rehabil..

[19] Andreani L., Ipponi E., Falcinelli F., Cordoni M., Bechini E., Vannucci L., D’Arienzo A., Capanna R. (2024). Proximal Femur Megaprostheses in Orthopedic Oncology: Evaluation of a Standardized Post-Operative Rehabilitation Protocol. Indian J. Orthop..

[20] Ziranu A., Bocchi M.B., Oliva M.S., Meschini C., Messina F., Calori S., Vitiello R. (2022). Survivorship of Proximal Femoral Replacement in Neoplastic and Non-Neoplastic Elderly Patients. Eur. Rev. Med. Pharmacol. Sci..

[21] Ruggieri P., Bosco G., Pala E., Errani C., Mercuri M. (2010). Local Recurrence, Survival and Function After Total Femur Resection and Megaprosthetic Reconstruction for Bone Sarcomas. Clin. Orthop..

[22] Pitera T., Guzik G., Biega P. (2017). Assessment of Post-Operative Physical Performance in Patients after Resection Arthroplasty of the Proximal Femur. Ortop. Traumatol. Rehabil..

[23] Andreani L., Ipponi E., Falcinelli F., Barderi S., Vannucci L., Campo F.R., D’Arienzo A., Parchi P.D. (2023). Distal Femur Megaprostheses in Orthopedic Oncology: Evaluation of a Standardized Post-Operative Rehabilitation Protocol. Healthcare.

[24] Fearon K., Strasser F., Anker S.D., Bosaeus I., Bruera E., Fainsinger R.L., Jatoi A., Loprinzi C., MacDonald N., Mantovani G. (2011). Definition and Classification of Cancer Cachexia: An International Consensus. Lancet Oncol..

[25] Brown L.R., Thomson G.G., Gardner E., Chien S., McGovern J., Dolan R.D., McSorley S.T., Forshaw M.J., McMillan D.C., Wigmore S.J. (2024). Cachexia Index for Prognostication in Surgical Patients with Locally Advanced Oesophageal or Gastric Cancer: Multicentre Cohort Study. Br. J. Surg..

[26] Denissen J.J.P.M., Koenders N., van Hinte G., Groen F., van der Wees P.J., van der Geest I.C.M., Dierselhuis E.F. (2023). Functional Outcomes after Reverse Shoulder Megaprosthesis Following Resection of Malignant Bone Tumor in the Proximal Humerus: A Systematic Review and Meta-Analysis. JSES Int..

[27] Trung D.T., Tran Q., Tu N.V., Quang S.N.T., Huu M.N., Trung H.P. (2021). Non-Oncologic Indication for Elbow Megaprothesis Replacement: 2 Cases Report. Int. J. Surg. Case Rep..

[28] Vaishya R., Thapa S.S., Vaish A. (2020). Non-Neoplastic Indications and Outcomes of the Proximal and Distal Femur Megaprosthesis: A Critical Review. Knee Surg. Relat. Res..

[29] Richter A., Windhagen H., Ettinger M. (2021). Implantation of an Attachment Tube Preserves Knee Extension after Nonunion of Felix IV Fracture: A Case Report. J. Med. Case Reports.

